# Contrast enhanced delayed myocardial enhancement in patients with end stage liver cirrhosis – further evidence for cirrhotic cardiomyopathy

**DOI:** 10.1186/1532-429X-11-S1-P20

**Published:** 2009-01-28

**Authors:** Dirk Lossnitzer, Daniel Gotthardt, Stephanie Lehrke, Grigorios Korosoglou, Henning Steen, Peter Sauer, Evangelos Giannitsis, Hugo A Katus

**Affiliations:** University of Heidelberg, Heidelberg, Germany

**Keywords:** Dobutamine Stress, NTproBNP Level, Gadopentetate Dimeglumine, Alcoholic Liver Cirrhosis, Patient Undergo Liver Transplantation

## Introduction

Cirrhotic cardiomyopathy was first described in 1969 by Regan TJ et al. and was found in all kinds of liver cirrhosis since 1990. Nevertheless the disease is still not well understood due to the hyperdynamic LV function, the lack of reliable diagnostic tools as well as multimorbidity of patients with end stage liver disease. However there is evidence for a reduced cardiac response to stress factors, QT-prolongation as well as myocardial fibrosis, subendocardial edema and vacuolization of myocytes.

## Purpose

The aim of this study was to further investigate the evidence of cirrhotic cardiomyopathy in patients with end stage liver disease by cardiac MRI.

## Methods

We examined 20 patients (8 females, age 54 ± 9) who are/were listed for liver transplantation (MELD score 15 ± 6, CHILD score 8.7 ± 2) because of alcoholic liver cirrhosis on a 1.5 T Philips Achieva scanner using a 5-element phased array cardiac synergy coil in 2007. Multiple short axes (SAX) cine images using a SSFP sequence with parallel imaging were acquired for the assessment of left ventricular ejection fraction (LVEF %) followed by a standardized high-dose dobutamine stress protocol up to 40 μg per kilogram of body weight per minute. A 3D FFE SAX multi-slice inversion recovery sequence 10 minutes post-Gd (Gd = 0.2 mmol/kg of gadopentetate dimeglumine (Schering, Berlin, Germany)) was employed for the measurement of LE. TNT and NT-proBNP were measured according to clinical routine procedures (Roche Diagnostics, Penzberg, Germany).

## Results

**Figure 1:**
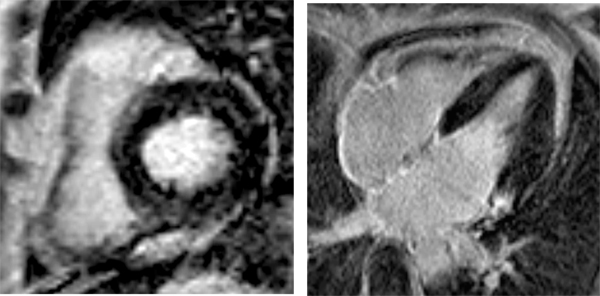
Figure 1

All patients showed normal to hyperdynamic LVEF at rest (71,3 ± 8%) as described before with other imaging methods and no wall motion abnormalities at rest or during dobutamine stress. However relevant LE (27,4 ± 17,4% of myocardial mass (see figure 1), was detected in all patients. NTproBNP levels were increased (846 ± 1042 pg/ml), but there was no correlation between the amount of LE and the NTproBNP levels (r = -0.04). TNT levels were within normal range (0,0225 ± 0,008 ng/ml). 8 patients underwent liver transplantation and 5 patients (3 of them after liver transplantation) died during the study period.

## Conclusion

This is the first description of myocardial delayed contrast enhancement in patients with end stage liver disease. We assume that these findings might be of diagnostic and prognostic value and might be helpful to detect patients at risk for cardiac complications on the waiting list for liver transplantation. However further studies with bigger sample sizes have to be done to fortify our results.

